# Canine leishmaniasis in the Americas: etiology, distribution, and clinical and zoonotic importance

**DOI:** 10.1186/s13071-024-06282-w

**Published:** 2024-04-30

**Authors:** Filipe Dantas-Torres

**Affiliations:** Aggeu Magalhães Institute, Fundação Oswaldo Cruz (Fiocruz), Recife, Brazil

**Keywords:** Americas, Dogs, Phlebotomine sand flies, *Leishmania*, *Lutzomyia*, Zoonosis

## Abstract

Canine leishmaniasis is a widespread disease on the American continent, with cases reported from Uruguay to the USA and Canada. While numerous *Leishmania* spp. have been reported in dogs in this region, *Leishmania infantum* and *Leishmania braziliensis* are the most common etiological agents of canine leishmaniasis from a continental perspective. Nonetheless, other species may predominate locally in some countries. The participation of dogs in the transmission cycle of various *Leishmania* spp. has long been speculated, but evidence indicates that their role as reservoirs of species other than *L. infantum* is negligible. Various native wildlife (e.g., small rodents, marsupials, sloths, and monkeys) are, in fact, the primary hosts of *Leishmania* spp. in the Americas. In this review, an updated list of *Leishmania* spp. infecting dogs in the Americas is presented, along with their distribution and clinical and zoonotic importance

**Graphical Abstract:**

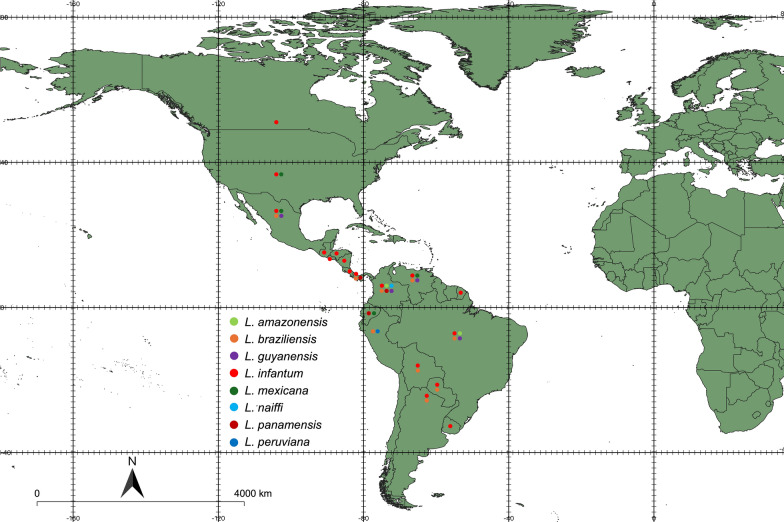

## Background

Canine leishmaniasis is one of the most important vector-borne diseases affecting dogs worldwide [1]. This disease is caused by parasites of the genus *Leishmania* (Kinetoplastida: Trypanosomatidae), which are primarily transmitted by phlebotomine sand flies (Diptera: Psychodidae: Phlebotominae) [2]. Globally, *Leishmania infantum* is the most common species involved in the etiology of canine leishmaniasis [1]. *Leishmania infantum* infection in dogs may range from subclinical to life threatening, depending on the host’s ability to mount an effective immune response to the intracellular forms of the parasite [3]. In addition to the clinical importance, dogs are the most important reservoir hosts in the zoonotic transmission cycle of *L. infantum* [4, 5], which further highlights the importance of this disease from a public health perspective.

Although its clinical and zoonotic importance is beyond debate, *L. infantum* is not the only species involved. In fact, several other zoonotic *Leishmania* spp. have been detected in dogs worldwide [6–8], particularly in the Americas [9, 10]. In more recent years, the detection of distinct *Leishmania* spp. in dogs has been facilitated by the application of advanced DNA sequencing methodologies, including next-generation sequencing and nanopore sequencing [11, 12].

The objective of this review is to update and expand upon a previous review of canine leishmaniasis in South America [9]. The list of species infecting dogs is updated and extended to the whole American continent. An updated distribution map is provided, and the role of dogs as reservoirs of various *Leishmania* spp. on this continent is also discussed.

## Etiology of canine leishmaniasis in the Americas

Numerous *Leishmania* spp. have been reported in dogs in the Americas (Table 1), including *Leishmania amazonensis*, *Leishmania braziliensis*, *Leishmania colombiensis*, *Leishmania guyanensis*, *Leishmania infantum*, *Leishmania mexicana*, *Leishmania panamensis*, *Leishmania peruviana*, *Leishmania pifanoi*, and *Leishmania naiffi* [9, 11–18]. Among these, *L. colombiensis* and *L. pifanoi* should be removed from the list of *Leishmania* spp. infecting dogs, as discussed below.

**Table 1:** *Leishmania* spp. reported in dogs in the Americas, with their proven or suspected animal reservoirs and sand fly vectors

*Leishmania* spp.	Proven or suspected wild reservoirs ^a^	Proven or suspected vectors ^b^	Geographical distribution (in dogs)
*L. amazonensis*	Several species of small rodents, opossums, and other wildlife	*Bichromomyia flaviscutellata*, *Bichromomyia olmeca olmeca*, and * Pintomyia nuneztovari*	Brazil [43, 44] and Colombia [15, 92]
*L. braziliensis*	Several species of small rodents, opossums, and other wildlife	*Nyssomyia intermedia*, *Nyssomyia neivai*, *Nyssomyia whitmani*, *Migonemyia migonei*, *Psychodopygus complexus*, *Psychodopygus davisi*, and * Psychodopygus wellcomei*	Argentina [93], Bolivia [94], Brazil [95], Colombia [15, 45], Mexico [96], Panama [13], Paraguay [97] ^c^, Peru [65], and Venezuela [98]
*L. guyanensis*	Linnaeus’s two-toed sloth (*Choloepus didactylus*), southern tamandua (*Tamandua tetradactyla*), and other wildlife	*Nyssomyia anduzei* and *Nyssomyia umbratilis*	Brazil [17], Colombia [14] and Venezuela [99]
*L. infantum*	Several species of carnivores (mainly canids), rodents, opossums, monkeys, and other wildlife	*Lutzomyia cruzi*, *Lutzomyia longipalpis*, *Migonemyia migonei*, *Pintomyia evansi*, and *Psathyromyia shannoni*	Argentina [100], Bolivia [94], Brazil [101], Canada [102] ^d^, Colombia [15], Costa Rica [26], El Salvador [26], French Guiana [16], Guatemala [26], Honduras [26], Mexico [26], Nicaragua [26], Panama [18] ^e^, Paraguay [26], United States [86, 102, 103], Uruguay [33], and Venezuela [104]
*L. mexicana*	Several species of small rodents, opossums, and other wildlife	*Bichromomyia olmeca olmeca*, *Dampfomyia anthophora*, and * Lutzomyia diabolica*	Ecuador [21], Mexico [105, 106], United States [107], and Venezuela [99]
*L. naiffi*	Nine-banded armadillo (*Dasypus novemcinctus*), Paraguayan punaré (*Thrichomys pachyurus*), and São Lourenço punaré (*Thrichomys laurentius*)	*Lutzomyia gomezi*, *Lutzomyia tortura*, *Nyssomyia trapidoi*, *Psychodopygus amazonensis*, *Psychodopygus ayrozai*, *Psychodopygus paraensis*, and *Psychodopygus squamiventris*	Colombia [11]
*L. panamensis*	Hoffmann’s two-toed sloth (*Choloepus hoffmanni*) and other wildlife	*Lutzomyia gomezi*, *Nyssomyia trapidoi*, *Nyssomyia ylephiletor*, and *Psychodopygus panamensis*	Colombia [45], Ecuador [108], and Panama [13, 109] ^f^
*L. peruviana*	Andean white-eared opossum (*Didelphis pernigra*) and Andean pericote (*Phyllotis andium*)	*Lutzomyia peruensis* and *Pintomyia verrucarum*	Peru [65]

^a^ Based on Maia et al. [60] and Roque et al. [61]. Listed animal species are not necessarily present or suspected reservoirs in the country where dogs have been found to be infected

^b^ Based on Lainson [22], Brazil et al. [90], and Cantanhêde et al. [91]. Alphabetically listed sand fly species are not necessarily proven suspected vectors in all countries where dogs have been found to be infected

^c^ Reported at the Royal Society of Tropical Medicine and Hygiene Meeting at Manson House, London, 13 December 2001 (Oddone et al. [97]; personal communication on 15 April 2024)

^d^ Cases in dogs with no travel history outside of Canada; it is uncertain if the dogs were infected through vertical, direct, or vector‐borne autochthonous transmission [102]

^e^ Supposedly imported cases [18] but autochthonous vectorial transmission is likely, as *Lu. longipalpis*, the main vector of *L. infantum*, is present in Panama [81]

^f^ Herrer and Christensen [13] obtained nine isolates from dogs, which were initially identified as *L. braziliensis* based on hamster pathogenesis. Later, Christensen et al. [109] concluded that these were *L. panamensis* (reported as “*Leishmania braziliensis panamensis*”)

In 1990, Hashiguchi et al. [19] reported an isolate (MCAN/EC/88/INU2) identified as *L. pifanoi* obtained from a dog in Paute, Ecuador. In their original report, they provided no detailed information on the methods used for species identification. They said that the “INU 2 strain from Paute […] identified as *Le. pifanoi* (Tesh and Grimaldi, personal communication)” and “Detailed characterization of these *Leishmania* isolates from animals will be published elsewhere.” Previous review articles have cited this report of *L. pifanoi* in a dog from Ecuador [9, 10, 20]. Nonetheless, I recently dug a little deeper into the literature and found that the species reported by Hashiguchi et al. [19] as *L. pifanoi* was, in fact, *L. mexicana*, as they reported 1 year later, based on more comprehensive analyses, including restriction endonuclease analysis of *Leishmania* kinetoplast DNA [21]. *Leishmania pifanoi* is indeed very similar to *L. mexicana* (in fact, considered as synonyms by some authors), which probably resulted their initial misidentification by using monoclonal antibodies, isoenzyme electrophoresis, or both (unclear in Hashiguchi et al. [19]). Nonetheless, *L. pifanoi* is a species apparently restricted to Venezuela [22]. As a curiosity, my previous difficulty in finding this information in literature resulted from the fact that Hashiguchi et al. [21] slightly changed the strain name from MCAN/EC/88/INU2 (as reported originally in Hashiguchi et al. [19]) to MCAN/EC/88/PauteInu2.

*Leishmania colombiensis* is another species previously reported in dogs that should be excluded from the list of causative agents of canine leishmaniasis in the Americas. This species was reported in a dog from Venezuela that showed clinical signs of visceral leishmaniasis [23]. It is difficult to determine whether this parasite was the cause of the disease in the dog, and coinfection with *L. infantum*, which is also present in Venezuela, cannot be ruled out. More importantly, that species has recently been transferred to the genus *Endotrypanum* [24], so it should no longer be considered an agent of leishmaniasis in the narrow sense. Nonetheless, further investigations are needed to determine whether this parasite is pathogenic to dogs. If proven, the disease caused by *E. colombiensis* should be named canine endotrypanosomosis. Interestingly, this parasite is reputed to cause both cutaneous and visceral diseases in humans [25].

Other *Leishmania* spp. reported in humans but not in dogs in the Americas include *L. lainsoni*, *L. lindenbergi*, *L. shawi*, and *L. venezuelensis* [22, 26].

## Geographical distribution

**Fig. 1:**
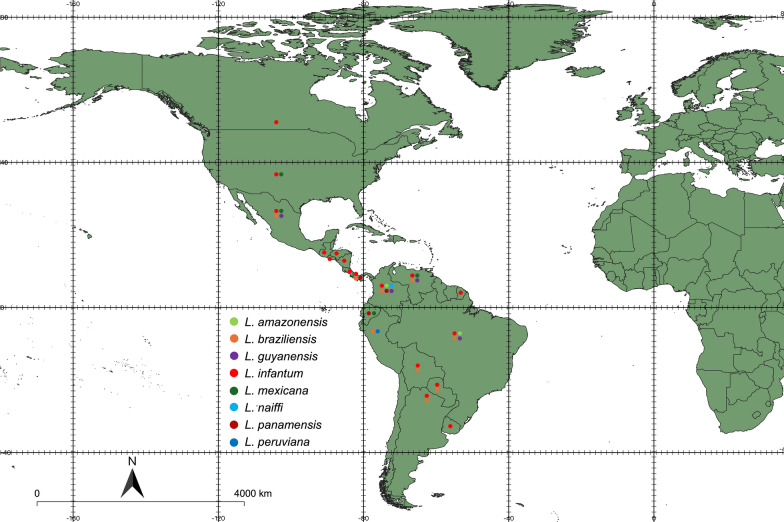
Country-by-country distribution of *Leishmania* spp. in dogs in the Americas. This map was constructed with QGIS (https://qgis.org/en/site) and Natural Earth (https://www.naturalearthdata.com)

Available data indicate that canine leishmaniasis is widespread on the American continent, with cases described from Uruguay to the USA and Canada (Fig. 1) (Table 1). Across this wide distribution range, canine leishmaniasis is considered absent or rare in some countries, such as Belize, Chile, Guyana, and Suriname. However, there is enough evidence (e.g., the presence of sand fly vectors, human cases, and/or seropositive dogs) indicating the risk of canine leishmaniasis in these countries [27–30]. For instance, leishmaniasis is considered nonendemic in Chile, but supposedly, imported cases in humans [31] and seropositive dogs [29, 30] have been reported. Little is known about the sand fly fauna of Chile, where only a single sand fly species (*Oligodontomyia isopsi*) has been reported thus far [32]. Nonetheless, additional field studies are necessary to obtain a better picture of canine leishmaniasis in these countries, particularly to identify the causative agent and to confirm the presence of putative vectors.

While canine leishmaniasis is not a notifiable disease in the Americas, it is reasonable to suppose that it is present in areas where human cases have been reported. As of 2012, human cases of visceral leishmaniasis have been reported in Argentina, Bolivia, Colombia, El Salvador, Honduras, Mexico, Nicaragua, Paraguay, and Venezuela [26]. Uruguay has now been added to this list, as both canine [33] and human cases [34] have been reported. French Guiana is still outside of this list, as no human cases of visceral leishmaniasis have been officially reported. Nonetheless, autochthonous cases of *L. infantum* infection in dogs have now been described in French Guiana [16], 13 years after a supposedly imported case [35]. This suggests that human cases of visceral leishmaniasis may be underdiagnosed in French Guiana. A similar situation has been observed in Panama, where the presence of *Lutzomyia longipalpis* (the main vector of *L. infantum* in the Americas) has long been known [26]. Supposedly imported cases of *L. infantum* infection have been reported in dogs from Panama [18]; therefore, the risk of visceral leishmaniasis establishment in this country, if not yet established, is high.

Human cases of cutaneous leishmaniasis caused by various *Leishmania* spp. have been reported in Argentina, Belize, Bolivia, Brazil, Colombia, Costa Rica, El Salvador, Ecuador, French Guiana, Guatemala, Guyana, Honduras, Mexico, Nicaragua, Panama, Paraguay, Peru, Suriname, and Venezuela [26]. Information on the presence of dogs infected by *L. braziliensis* or other *Leishmania* spp. causing cutaneous leishmaniasis in some of these countries (e.g., Belize, Costa Rica, El Salvador, Guatemala, Guyana, Honduras, and Nicaragua) is limited or virtually nonexistent. Nonetheless, further investigations in some of these countries will likely reveal that canine infections caused by species such as *L. braziliensis* and *L. panamensis* are common.

*Leishmania infantum* and *L. braziliensis* are the most widespread agents of canine leishmaniasis in the Americas. Canine infections caused by *L. infantum* have been reported in 17 countries, whereas those caused by *L. braziliensis* have been described in nine countries (Table 1). The apparent absence of *L. braziliensis* infections dogs in some countries (e.g., Belize, Costa Rica, Ecuador, French Guiana, Guatemala, Honduras, and Nicaragua) may be due to the lack of published reports, as *L. braziliensis* is known to occur in humans in these countries. Infections with other species in dogs are apparently more restricted geographically, but considering their distribution in humans, canine cases may currently be underestimated. This may well be the case for *L. amazonensis*, *L. mexicana* and *L. panamensis*.

Data on the prevalence of *Leishmania* spp. infection in dogs in the Americas have been reviewed elsewhere (e.g., [9, 10, 20, 36]). While discussing prevalence studies is outside of my objective here, I just want to emphasize that prevalence is an indicator that may vary widely in space and time. For instance, a study reported data from 73,964 dogs screened from 2008 to 2017 by state public health authorities in the Sobral municipality (Ceará state, northeastern Brazil), a traditional focus of human visceral leishmaniasis endemicity [37]. Considering the whole study period, the mean seroprevalence in the municipality was 3.8%, ranging from 1.6% to 13.1% according to district. However, the seroprevalence in each district varied widely annually, surpassing 50% on several occasions. In a similar fashion, the seroprevalence may vary widely according to the test used, as concluded from studies conducted in areas of active cutaneous leishmaniasis transmission. According to a comprehensive review article published in 1999 [20], the mean seroprevalence values were estimated to be 32.1% (range 14.7–58.9%) and 16.6% (range 0–63.2%) using the enzyme-linked immunosorbent assay (ELISA, eight studies) and indirect immunofluorescence (21 studies), respectively. The mean percentage of dogs that were positive according to the Montenegro skin test (13 studies) was 25.5% (range 0–66.7%). Another issue concerning serological studies is the possible cross-reactivity between different *Leishmania* spp. and *Trypanosoma* spp., which may be common in some foci [38, 39].

While cross-sectional studies are commonly conducted in the Americas, particularly in Latin America (reviewed in [10, 36], longitudinal studies are rare [40, 41], probably due to the inherent difficulties pertaining to this type of study. A study conducted in Goiana (Pernambuco, northeastern Brazil) and São Joaquim de Bicas (Minas Gerais, southeastern Brazil) reported yearly crude incidences of 19.6% and 43.8%, respectively, which were estimated by both serology and PCR [41]. This means that every year, a relatively high proportion of seronegative dogs living in these areas will seroconvert, become PCR positive, or both. This type of information is very important for understanding disease dynamics in endemic foci and may help to determine the magnitude of the disease control problem [40].

## Clinical importance

From a clinical perspective, *L. infantum* is the agent of the most severe form of leishmaniasis in dogs [1, 42]. However, *L. amazonensis* has also been detected in dogs with clinical signs of visceral leishmaniasis [43, 44], which highlights the importance of using molecular approaches for a proper diagnosis and species identification. Excluding *L. infantum* and *L. amazonensis*, other *Leishmania* spp. have mostly been detected in dogs showing clinical signs of cutaneous leishmaniasis. While *L. braziliensis* is the most frequent agent of cutaneous leishmaniasis in dogs in the Americas, other species, such as *L. panamensis,* may also be common in some areas. For instance, during an outbreak of canine cutaneous leishmaniasis in Colombia, *L. panamensis* was isolated from 12 dogs, and *L. braziliensis* was isolated from eight dogs [45]. Regardless of the species involved, the dogs presented nodules or ulcers (0.4–10 cm in diameter), with evolution times ranging from 2 to 12 months [45]. Dogs presented single or multiple lesions but no systemic signs.

While canine cutaneous leishmaniasis is mostly a mild disease, some dogs may present disfiguring mucosal lesions and may sometimes die due to other health complications [46]. In Yucatan, Mexico, a 10-year-old intact female Chihuahua with cutaneous leishmaniasis attributed to *L. mexicana* died, probably due to renal failure (urea, 157 mg/dL; creatinine, 4 mg/dL) [46]. This dog was already receiving ramipril and furosemide due to congestive heart failure [46]. It is unlikely that *L. mexicana* infection itself caused this clinical condition, resulting in patient death, and information on the real-time PCR assay employed for species identification is incomplete. Nonetheless, although cutaneous leishmaniasis in dogs is usually a mild disease, this case highlights that a complete clinical evaluation of dogs with cutaneous leishmaniasis may be important, especially for geriatric dogs or dogs with other underlying medical conditions which could worsen the prognosis.

## Zoonotic importance

Dogs are the primary reservoirs of *L. infantum* in the Americas [4, 5]. In a study conducted on Marajó Island, Pará state, northern Brazil, the basic case reproduction number (*R*_0_) was estimated to be 5.9 [40]. This means that, on average, each infected dog could generate approximately six new cases. Nonetheless, the infectiousness may vary widely from dog to dog, with dogs with high parasite numbers in their skin generally being more infectious to phlebotomine sand fly vectors than dogs with lower parasite numbers [47, 48]. Cats [49] and several other wildlife species (e.g., wild canids and nonhuman primates) [50, 51] may also serve as sources of infection for phlebotomine sand flies, although their actual epidemiological importance in the zoonotic transmission cycle of *L. infantum* needs further investigation. A contemporary example of the role played by other animals as reservoirs of *L. infantum* comes from Spain, where hares and rabbits were identified as the main sources of infection to phlebotomine sand fly vectors during an outbreak of human leishmaniasis in Madrid [52]. A series of studies clearly demonstrated that dogs played no role in this outbreak [53]. A study demonstrated that *Lu. longipalpis* can pick up *L. infantum* amastigotes while feeding on asymptomatic humans and that sick individuals coinfected with human immunodeficiency virus (HIV) are more infectious to this vector [54]. Again, the role of infected humans in endemic foci needs to be better understood considering the potential consequences for the control of visceral leishmaniasis.

Although the role of dogs as reservoirs of *L. infantum* is unequivocal, the indiscriminate elimination of seropositive dogs (i.e., dog culling strategy) has not been successful in controlling the incidence of human visceral leishmaniasis in Brazil [55]. The ineffectiveness of this strategy has been attributed to several factors, including the existence of other reservoirs [55].

The role of dogs as reservoirs of other *Leishmania* spp., particularly *L. braziliensis*, has been extensively investigated in the Americas. Although dogs are frequently exposed to *L. braziliensis* in endemic areas, their participation in the zoonotic transmission cycle of this parasite is likely negligible [4, 20, 56–58]. Indeed, excluding *L. infantum*, which was introduced in the Americas [59], all other *Leishmania* spp. detected in dogs in the Americas are native to the Neotropical region and are primarily maintained by wildlife reservoirs [4, 60, 61]. For instance, small rodents are exceptional hosts for *L. braziliensis* [62–64].

Dogs have been repeatedly suggested as reservoirs of *L. peruviana* and *L. mexicana* in Peru and Ecuador, respectively [65]. However, these conclusions are based on weak circumstantial evidence, as reviewed elsewhere [4, 20]. Indeed, there are apparently no studies demonstrating the infectiousness of dogs infected by these parasites to their respective phlebotomine sand fly vectors. Moreover, both *L. peruviana* and *L. mexicana* have also been detected in a wide range of small mammal species, some of which are considered potential reservoirs [60, 65].

Similarly, there is no evidence suggesting that dogs are potential reservoirs of *L. panamensis* in Colombia, as discussed appropriately by Vélez et al. [45]. Reports of infection by *L. guyanensis* and *L. naiffi* in dogs are very rare and clearly incidental. For example, *L. naiffi* has only recently been found in humans and dogs [11, 66] in Colombia, where the wild animal reservoir is, in fact, unknown.

## Outstanding questions

Since 2009, an extraordinary number of field and laboratory studies on canine leishmaniasis in the Americas have been published in international literature. These include epidemiological studies focused on prevalence and risk factors (e.g., [67, 68]), studies validating new diagnostic tools (e.g., [69]), and clinical trials assessing the efficacy or effectiveness of therapeutic protocols (e.g., [70]) and prevention and control strategies (e.g., [71, 72]). The unified efforts of scientists, nongovernmental organizations [e.g., the Brasileish group (https://www.brasileish.com.br)], and public health authorities effectively contributed to positively changing our practices in terms of the diagnosis, treatment, prevention, and control of canine leishmaniasis, particularly in Brazil, where mass culling of seropositive dogs is no longer a common practice, 4% deltamethrin-impregnated dog collars are often applied to dogs in high-risk areas, and miltefosine is now licensed for use in dogs [55, 73, 74].

While most studies continue to be conducted by Brazilian leishmaniacs, the number of studies from other countries is also rapidly increasing (e.g., [18, 27–30, 33, 45]). It also amazes me the extraordinary number of studies on feline leishmaniasis, a neglected disease that is finally receiving the attention it deserves. These studies have, for instance, unequivocally demonstrated that cats are also infectious to sand fly vectors [49]. One of the outstanding research questions is the possible role of cats as reservoirs in the zoonotic transmission cycle of *L. infantum* [75]. The answer to this question may have practical implications for the control of leishmaniasis in areas where dogs, humans, and cats are at risk of infection.

Another important aspect to be understood is the unstoppable spread of canine leishmaniasis caused by *L. infantum* to new areas in the southern cone of South America [33] and to urbanized areas in already endemic regions [76]. The disease is also apparently expanding in the Caribbean region [77–80]. Drivers of this spreading process may include the movements of infected dogs and people and the expanding distribution range of sand fly vectors. For instance, *Lu. longipalpis*, which is present in Argentina, Bolivia, Brazil, Colombia, Costa Rica, El Salvador, Guatemala, Honduras, Mexico, Nicaragua, Panama, Paraguay, Uruguay, and Venezuela [81], is the main vector of *L. infantum* in most of the Americas. This vector has adapted to new areas in the southern cone of South America may be related to climate change [82]. In areas where *Lu. longipalpis* is absent, other species may be acting as local vectors of *L. infantum*. For instance, *Lutzomyia cruzi* is considered a vector of *L. infantum* in Corumbá city (Mato Grosso do Sul state, central-western Brazil) [83] and Bolivia [84]. Similarly, *Migonemyia migonei* is also a permissive vector of *L. infantum* and suspected to be involved in its transmission in some areas of Brazil [85] and Bolivia [84]. In the USA, *Psathyromyia shannoni* is a putative vector of *L. infantum* in areas where cases of canine leishmaniasis have been reported [86]. These examples suggest the need for more studies on the vectors involved in the *Leishmania* spp. transmission to dogs in the Americas, including in the USA [86].

Further research is needed to obtain a more reliable picture of the epidemiological situation of canine leishmaniasis in different American countries, where information is currently limited or virtually inexistent. Researchers should focus on the capture and identification of phlebotomine sand flies, detection of anti-*Leishmania* spp. antibodies, and molecular characterization of *Leishmania* spp. circulating in dogs. For instance, these studies could reveal the circulation of *L. braziliensis* among dogs in countries like Belize, Costa Rica, Ecuador, Guatemala, French Guiana, Honduras, and Nicaragua, where this parasite causes cutaneous and mucocutaneous leishmaniasis in humans [26].

## Conclusions

Canine leishmaniasis is a widespread disease in the Americas, with a seroprevalence exceeding 50% in highly endemic foci. The disease may be caused by different *Leishmania* spp., but *L. infantum* and *L. braziliensis* are the most widespread and prevalent from a continental perspective. *Leishmania infantum* clearly expanded southwards in recent decades and is now endemic to parts of the southern cone of South America, including Uruguay. Considering the clinical importance of canine leishmaniasis and the limited treatment availability in the Americas, the use of preventive measures [55, 72–74, 87–89] is key to mitigating the risk of infection in uninfected dogs. This may also minimize the role of infected dogs as reservoirs, which is pivotal to reduce the risk of infection in humans and other susceptible animals, including cats.

## Data Availability

All the data supporting the conclusions of this review are cited in the references.
